# Viral S protein histochemistry reveals few potential SARS-CoV-2 entry sites in human ocular tissues

**DOI:** 10.1038/s41598-021-98709-y

**Published:** 2021-09-27

**Authors:** Gottfried Martin, Julian Wolf, Thabo Lapp, Hansjürgen T. Agostini, Günther Schlunck, Claudia Auw-Hädrich, Clemens A. K. Lange

**Affiliations:** Eye Center, Medical Center, Medical Faculty, University of Freiburg, Killianstr. 5, 79106 Freiburg, Germany

**Keywords:** Molecular medicine, Infection

## Abstract

Despite the reported low expression of the primary SARS-CoV-2 receptor ACE2 in distinct ocular tissues, some clinical evidence suggests that SARS-CoV-2 can infect the eye. In this study, we explored potential entry sites for SARS-CoV-2 by viral S protein histochemistry on various ocular tissues and compared the staining patterns with RNA and protein expression of TMPRSS2 and ACE2. Potential viral entry sites were investigated by histochemistry using tagged recombinant viral S protein on 52 ocular tissue samples including specimens of the cornea, conjunctiva, lid margin, lacrimal gland tissue, retina, choroid, and RPE. In addition, ACE2 and TMPRSS2 immunohistochemistry were performed on the same ocular tissue, each with distinct antibodies binding to different epitopes. Lung tissue samples were used as positive controls. Finally, bulk RNA sequencing (RNA-Seq) was used to determine the expression of ACE2 and its auxiliary factors in the tissues mentioned above. S protein histochemistry revealed a positive staining in lung tissue but absent staining in the cornea, the conjunctiva, eye lid samples, the lacrimal glands, the retina and the optic nerve which was supported by hardly any immunoreactivity for ACE2 and TMPRSS2 and scarce *ACE2* and *TMPRSS2* RNA expression. Negligible staining with antibodies targeting ACE2 or TMPRSS2 was seen in the main and accessory lacrimal glands. In contrast, ocular staining (S protein, ACE2, TMPRSS2) was distinctly present in pigmented cells of the RPE and choroid, as well as in the ciliary body and the iris stroma. S protein histochemistry revealed hardly any SARS-CoV-2 entry sites in all ocular tissues examined. Similarly, no significant ACE2 or TMPRSS2 expression was found in extra- and intraocular tissue. While this study suggest a rather low risk of ocular infection with SARS-CoV-2, it should be noted, that potential viral entry sites may increase in response to inflammation or in certain disease states.

## Introduction

SARS-CoV-2 (severe acute respiratory syndrome coronavirus 2) is a novel human coronavirus that sparked a pandemic in late 2019 posing a major threat to human health. Similar to other coronaviruses, the main entry site of SARS-CoV-2 into the human body is through the respiratory tract, as it cannot penetrate an intact skin-barrier. Nevertheless, there is an ongoing debate if SARS-CoV-2 also can enter the human body via ocular tissues such as the cornea, the conjunctiva, or the lacrimal duct^1–3^. The latter ends within the nose and would allow an infection via the respiratory tract. Viral load detected in conjunctival or corneal swabs of COVID-19 patients by PCR was much lower than that found in nasopharyngeal or sputum specimen^4^. The prevalence was reported to be 1.6 to 7.1%^5^, 0 to 29%^6^, or 0 to 7.4%^4^.

SARS-CoV-2 uses its viral S protein to bind to ACE2 (angiotensin-converting enzyme 2), the main receptor protein to enter cells^7,8^. Afterwards, the S protein is cleaved by TMPRSS2 (transmembrane protease serine 2) resulting in membrane fusion of the virus with the host cell^9^. Therefore, a tissue that can be infected by SARS-CoV-2 is expected to express ACE2 and TMPRSS2 or other co-receptors such as BSG (basigin, CD147) or NRP1 (neuropilin 1). There are conflicting data in the literature on the expression levels of ACE2 and TMPRSS2 in ocular tissue. While some studies suggest an expression of both proteins in ocular tissues^10–15^ others reported negligible expression levels^16–18^. This discrepancy may be explained by different methods and antibodies used in these publications, resulting in a variety of weak to intense staining patterns. This contradictory information is in urgent need of clarification. In the present study, we used biotin-tagged recombinant viral S protein for histochemical staining and compared it to staining patterns of antibodies raised against ACE2 or TMPRSS2, each with distinct antibodies binding to different epitopes. The results of our study reveal hardly any staining by S protein histochemistry and no significant ACE2 or TMPRSS2 expression in all ocular tissues examined, suggesting a rather low risk of ocular infection with SARS-CoV-2.

## Materials and methods

### Human ocular specimens

For immunohistochemistry, 8 corneal specimens (from whole eyes enucleated for choroidal melanoma), 8 healthy conjunctival specimens (no conjunctivitis, obtained with retinal ablation surgery), 8 specimens from the lid (obtained from ectropion or entropium repair), 10 specimens from the lacrimal gland (obtained in orbital and lid mass removal), and 18 whole eyes with various diagnoses (normal, trauma, circulatory disorders, glaucoma, retinal detachment) were prepared and analysed. Lung tissue served as positive controls.

For RNA sequencing (RNA-Seq), 8 healthy conjunctival specimens (no conjunctivitis, obtained with retinal ablation surgery), 34 diseased conjunctival specimens (comprised of 12 melanoma, 7 squamous cell carcinoma, 7 papilloma, 8 pterygia), 8 healthy lacrimal gland specimens (obtained in orbital and lid mass removal), 3 nasal mucosa specimens (obtained with dacryocystorhinostomy), 4 choroid/RPE (retinal pigment epithelium) and 6 retina specimens (from whole eyes enucleated for choroidal melanoma) were included.

In accordance with the ethical requirements, the analysis of all tissue samples was performed in an anonymized manner. Institutional Review Board (IRB) as well as approval of the Ethics Committee of the Albert-Ludwigs-University Freiburg was obtained for specimen acquisition, the use of human tissue samples for scientific analysis, as well as data generation and presentation. Informed consent was obtained from all patients and/or their legal guardians.

### Tissue fixation and preparation

Formalin fixation and paraffin embedding (FFPE) of ocular samples were performed immediately after tissue excision according to routine protocols, as previously described^16^. Following routine histological staining, each specimen’s histological diagnosis was made by two experienced ophthalmic pathologists. For RNA sequencing, fifteen 4 µm thick FFPE sections were collected and stored in tubes prior to RNA extraction.

### RNA-Seq

RNA isolation from FFPE specimens was carried out as previously described^16^. Briefly, total RNA was extracted from FFPE samples using the Quick-RNA FFPE Kit (Zymo Research, Irvine, California). Following DNAse I digestion using the Baseline-ZERO Kit (Epicentre, Madison, Wisconsin), the RNA concentration was quantified using the Qubit RNA HS Assay Kit on a Qubit Fluorometer (Life Technologies, Carlsbad, California). RNA quality was determined via the RNA Pico Sensitivity Assay on a LabChip GXII Touch (PerkinElmer, Waltham, Massachusetts). RNA sequencing was performed using massive analysis of cDNA ends (MACE), a 3’ RNA sequencing method, as previously described^19^. The barcoded libraries comprising unique molecule identifiers were sequenced on the NextSeq 500 (Illumina, USA) with 1 × 75 bp. PCR bias was removed using unique molecular identifiers.

Sequencing data were uploaded to and analyzed on the Galaxy web platform (https://usegalaxy.eu)^20^ as previously described^16^. Quality control was performed with FastQC Galaxy Version 0.72 (http://www.bioinformatics.babraham.ac.uk/projects/fastqc last access on 08/10/2020). Reads were mapped to the human reference genome (Gencode 34, https://www.gencodegenes.org/human/releases.html) with RNA STAR Galaxy Version 2.7.5b^21^ (default parameters) using the Gencode annotation file (Gencode 34). Reads mapped to the human reference genome were quantified using featureCounts Galaxy Version 1.6.4^22^ (default parameters). The output of featureCounts was imported to RStudio (Version 1.2.1335, R Version 3.5.3). Transcripts per million were calculated based on the output of featureCounts (assigned reads and feature length), as previously described^23^. Gene symbols and gene types were defined based on ENSEMBL release 101 (Human genes, download on 11/23/2020)^24^. Transcripts per million for *ACE2*, *TMPRSS2*, *NRP1*, *ACTA2* (actin alpha 2, smooth muscle), *BSG*, *FURIN*, *DPP4*, *CTSL*, *HSPA5*, *ANPEP*, *CEACAM1*, *CD209* and *CLEC4M* were extracted from the data and plotted as boxplots using ggplot2^25^. The sequencing raw data are available in the Gene Expression Omnibus Database under the following accession numbers: GSE148387 (conjunctiva and conjunctival melanoma), GSE149004 (conjunctiva, conjunctival carcinoma and papilloma), GSE155776 (pterygium), GSE159358 (lacrimal gland), GSE159357 (retina) and GSE146887 (choroid/RPE).

### Immunohistochemistry

Human ocular FFPE specimens were processed according to established standard protocols in the ophthalmopathological department of the Eye Center, University Medical Center in Freiburg. After demasking the sections in boiling Tris–EDTA buffer for 30 min, staining was done by standard procedures as previously described^16^. Briefly, after blocking with 2 drops of Ultrablock (UltraVision Protein Block, Thermo Scientific, Germany), primary antibodies (100 µl, see Table 1) were incubated for 1 h at room temperature (RT) followed by 100 µl of biotinylated secondary antibodies applied for 30 min at RT (donkey anti-mouse #715-065-151 and donkey anti-rabbit #711-065-152, Jackson ImmunoResearch, Ely, UK). Next, one drop of streptavidin coupled with alkaline phosphatase for 30 min at RT (#5550-0002, SeraCare, Milford, MA, USA), and 150 µl of Vector Red AP Substrate Kit I for 8 min at RT (#SK-5100, Vector Labs, Burlingame, CA, USA) resulting in red staining. Sections were counter-stained with hematoxylin. In some cases, biotin blocking (Avidin/Biotin Blocking Kit, #ab64212, Abcam) was applied after blocking with Ultrablock. Dilution of the antibodies was varied to determine the appropriate antibody concentration for optimal staining and to check for specificity (Suppl. Figure 1). ACE2-ab15348 and TMPRSS2-HPA035787 showed a good signal-to-noise ratio whereas TMPRSS2-ab109131 had a tendency for background. ACE2-AMAB91262 showed weak staining with very little background.

**Table 1:** Antibodies.

Protein	Host	AB type	Clone	Epitope	Supplier	Code	Dilution
ACE2	Mouse	Monoclonal	171,606	whole protein	Biotechne	MAB933	1:50
ACE2	Mouse	Monoclonal	CL4035	N-terminal	Sigma	AMAB91262	1:500 or 1:2000
ACE2	Rabbit	Polyclonal		whole protein	Sigma	HPA000288	1:500
ACE2	Rabbit	Polyclonal		C-terminal	Abcam	ab15348	1:1000
TMPRSS2	Rabbit	Monoclonal	EPR3862	C-terminal	Abcam	ab109131	1:2000
TMPRSS2	Rabbit	Polyclonal		N-terminal	Sigma	HPA035787	1:250
ACTA2	Mouse	Monoclonal			Sigma	A2547	1:2000
AIF1	Rabbit	Monoclonal	EPR16588		Abcam	ab178846	1:4000
CD68	Mouse	Monoclonal	PG-M1		Dako	M0876	1:50

Antibodies #MAB933 and #HPA000288 were produced using the same antigen.

Staining with viral S protein as primary detection agent was performed by a similar procedure and established in lung tissue. Paraffin sections were demasked in Tris–EDTA buffer as above and blocked in TBST (Tris-buffered saline with 0.1% Tween 20) containing 2% BSA (bovine serum albumin). S protein (Biotinylated SARS-CoV-2 S protein receptor binding domain (RBD), #SPD-C82E9, Acro Biosystems, Newark, DE, USA, 200 µg/ml) was diluted 1:500 in TBST and sections were incubated in 100 µl S protein solution for 1 h at RT. Streptavidin-coupled alkaline phosphatase and Vector Red AP substrate were applied as above. During the set-up for S protein staining, the amount of BSA appropriate for blocking was verified with sections from lung tissue (Suppl. Figure 2).

## Results

### S protein and ACE2 immunoreactivity on the ocular surface

**Figure 1:**
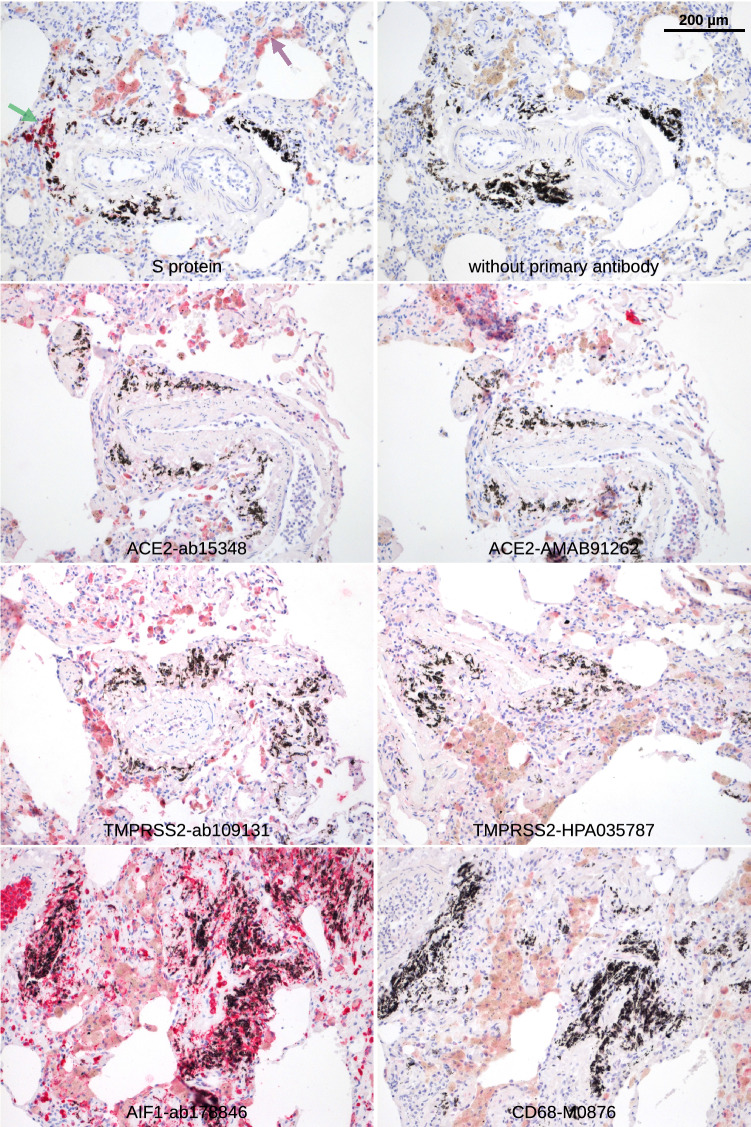
Lung. Paraffin sections of normal human lung were stained with S protein or different antibodies as indicated. Red staining was found in pigmented cells (macrophages that are positive for AIF1 and CD68, purple arrow) as well as cells at black material that are probably also a subtype of macrophages (green arrow).

**Figure 2:**
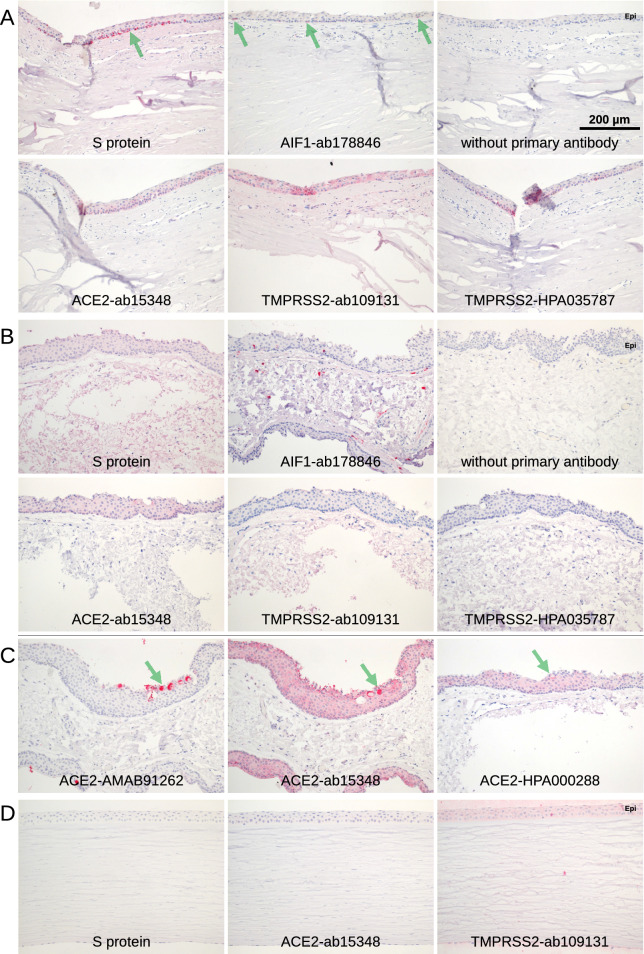
Conjunctiva and cornea. Immunohistochemical staining of serial paraffin sections. (**A**) Staining (red) of the basal part of the epithelium of limbal conjunctiva was observed in a single specimen (green arrows). The staining was very distinctive with S protein but less distinctive for the TMPRSS2-HPA035787 antibody while ACE2-ab15348 and TMPRSS2-ab109131 showed a broader pattern over the whole thickness of the epithelium. The staining was restricted to the area shown and not found at the other side of the cornea. Staining for AIF1 showed a comparable pattern indicating that the cells are macrophages. (**B**) Conjunctiva. S protein is overstained with uniform staining of every tissue. The epithelium (Epi) may be stained with antibody ACE2-ab15348. Red staining for AIF1 showed some epithelial and sub-epithelial macrophages. (**C**) Staining of goblet cells was found at single sites of some of the conjunctival specimen with antibodies raised against ACE2 only (green arrows). ACE2-ab15348 showed an additional staining in the epithelium. (**D**) Only background staining was observed by S protein and antibodies for ACE2 and TMPRSS2 in cornea. Epithelium (Epi).

The ocular surface, including the cornea and conjunctiva, is directly exposed to the environment and provides a barrier to noxious stimuli and microorganisms, thus protecting the intraocular tissue and the vision apparatus. Histochemistry with the S protein showed a specific staining pattern in the lung tissue, which served as a positive control (Fig. 1), but no significant S protein staining in the cornea and conjunctiva. Only one single exception was found in the basal epithelial layer of the conjunctiva at the limbus of one eye (Fig. 2A, Table 2). These cells were also positive for AIF1 (allograft inflammatory factor 1, IBA1) indicating that they were macrophages. The same site was also stained by antibodies targeting ACE2 or TMPRSS2.

**Table 2:** Staining results.

Tissue or cells	S protein	ACE2	TMPRSS2
Cornea	0/8	Background only	Background only
Bulbar conjunctiva, macrophages	1/26	1/26	1/26
Bulbar conjunctiva, goblet cells	0/8	4/26, conjunctival specimens only	0/8
Tarsal conjunctiva, goblet cells	0/8	1/8	0/8
Tarsal conjunctiva, glandular tissue	0/8	Weak in 8/8	Background only
Lacrimal glands	0/10	Weak in 10/10	Background only
Nasal mucosa	0/3	Weak in 3/3	Background only
Iris	11/19	10/18	14/18
Ciliary body	17/19	15/18	17/18
Retina	0/19	11/18	14/18
RPE	15/19	18/18	18/18
Choroid	14/19	13/18	17/18

Indicated are numbers of specimens showing staining/total number of specimens.

Most of the conjunctival samples were negative for ACE2 (Fig. 2B). Only some showed a weak staining for ACE2 in the epithelium with the antibodies ACE2-ab15348 and ACE2-HPA000288. Epithelial staining of identical areas of the same sample varied from weakly positive to negative, indicating that the staining is near the detection limit. Interestingly, goblet cells were stained in distinct areas of some conjunctival specimens (4 out of 8, Table 2) with antibodies ACE2-ab15348, ACE2-HPA000288, and especially ACE2-AMAB91262 (Fig. 2C) but never with S protein. In every case, some groups of neighbouring goblet cells were stained while many others were not stained. Staining was concentrated in the goblet cell mucous.

**Figure 3:**
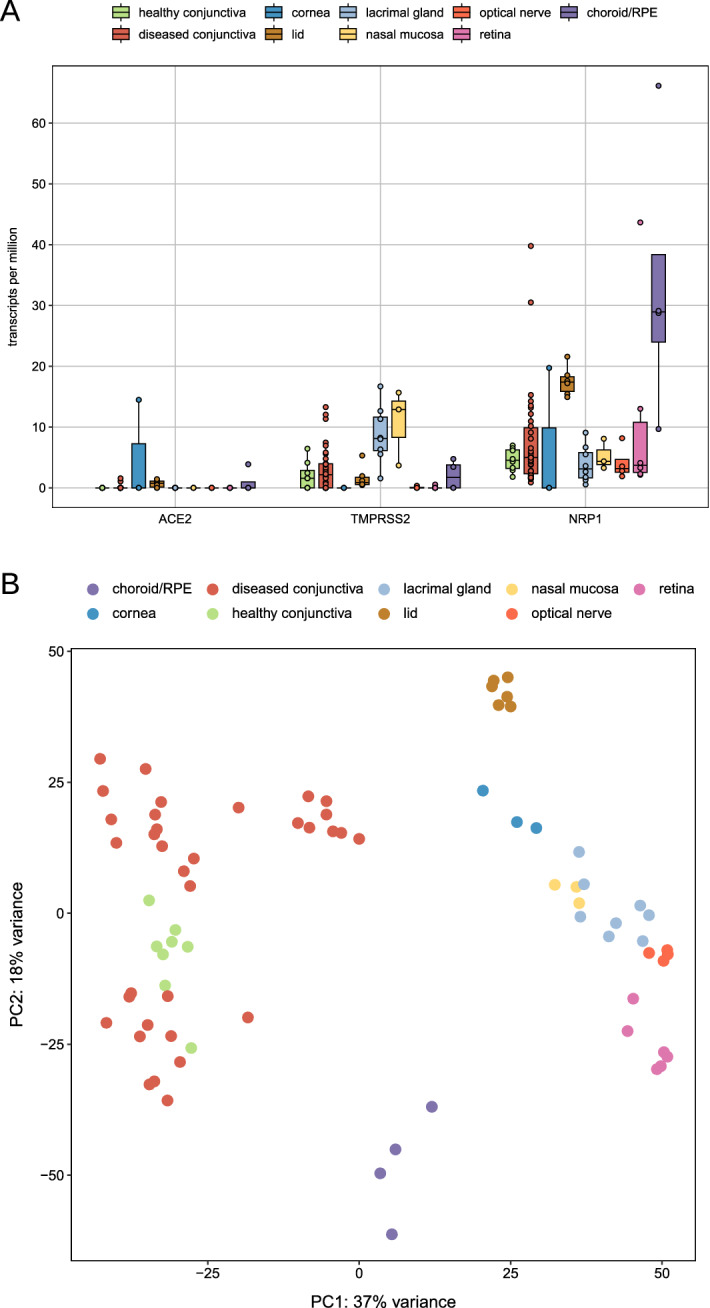
RNA sequencing. Boxplots showing ACE2, TMPRSS2 and NRP1 expression levels of all analyzed ocular samples (n = 63). Each dot represents one sample. Number of specimens: healthy conjunctiva: 8, diseased conjunctiva: 34, lacrimal gland: 8, nasal mucosa: 3, retina: 6, RPE/choroid: 4. *ACE2*: Angiotensin I Converting Enzyme 2, *TMPRSS2*: Transmembrane Serine Protease 2, *NRP1*: Neuropilin 1.

At RNA level, *ACE2* showed hardly any expression in 42 conjunctival samples (median 0.0 transcripts per million (TPM), interquartile range (IQR): 0.0–0.0, min 0.0 TPM, max 1.6 TPM). 95.2% of the samples (40) revealed no *ACE2* transcripts, and 2 of them negligible amounts of 1.1 and 1.6 TPM, respectively. A subgroup analysis of all conjunctival samples revealed that *ACE2* expression was not only insignificant in healthy conjunctival tissue (all samples 0 TPM) but also in samples of altered conjunctiva such as conjunctival papilloma (one sample with 1.6 TPM), squamous cell carcinoma (all samples with 0.0 TPM), melanoma (one sample with 1.1 TPM) or pterygium (all samples with 0.0 TPM). Similarly, conjunctival samples showed hardly any expression of *TMPRSS2* (median 2.1 TPM, IQR 0.0–4.0 TPM, min 0.0 TPM, max 13.3 TPM, samples with 0 TPM: 31.0%) (Fig. 3). As a reference, the expression of the conjunctival epithelial marker *KRT19* (keratin 19) was analyzed. The median expression for *KRT19* was 3902.3 TPM (IQR: 2277.6–5936.6) indicating a significant expression of this marker in the conjunctival samples analyzed (Suppl. Figure 3A).

In corneal tissue, no significant ACE2 staining was found in all analyzed sections (Fig. 2D). At RNA level, there was one sample with some *ACE2* expression (14.5 TPM), whereas two samples revealed 0 TPM. TPMRSS2 was not detectable in all 3 corneal specimens (all 0 TPM) (Fig. 3). As a reference, the expression of the *KRT5* (keratin 5) was 4487.0 TPM in median (IQR: 4471.0–5372.0) indicating a significant expression of this marker (Suppl. Figure 3A).

### S protein and ACE2 immunoreactivity in the lid, lacrimal gland and nasal mucosa

**Figure 4:**
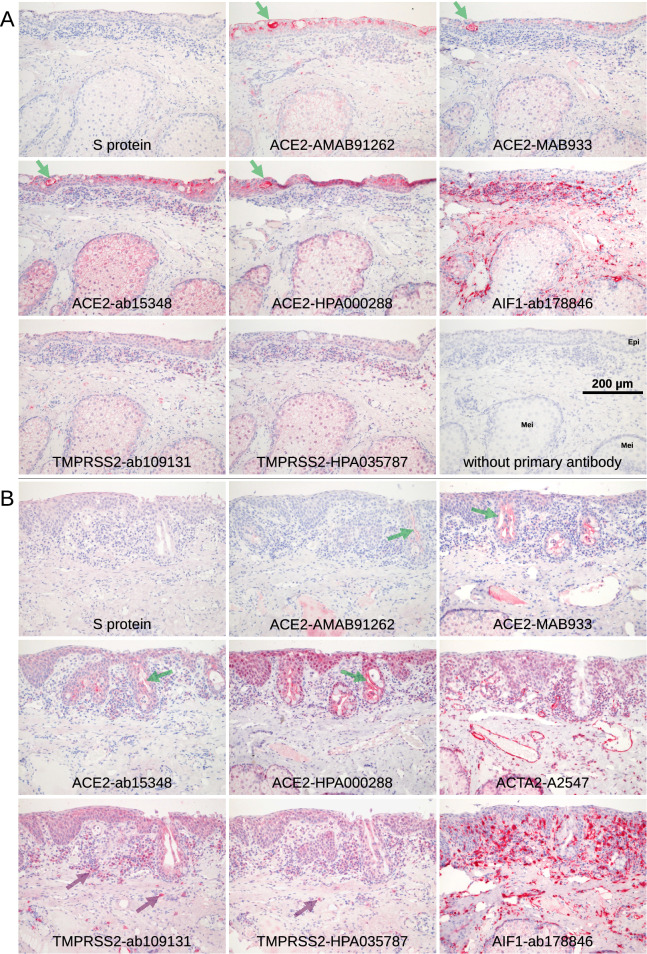
Eye lid. Serial paraffin sections were stained red with S protein or different antibodies raised against ACE2 or TMPRSS2. (**A**) Tarsal conjunctiva and Meibomian gland. S protein did not show any staining. A weak staining of the acinar cells of the Meibomian glands (Mei) was observed with all antibodies including AIF1 indicating that this weak staining is background. ACE2 and TMPRSS2 staining patterns differ. Their efferent ducts often showed a more intense staining in the extracellular sebaceous layer. Staining of the epithelium (Epi) was observed mainly in the tarsal part especially around Henle’s crypts. Staining of goblet cells within the epithelium (green arrows) was very rare (this case out of 8) and restricted to ACE2. (**B**) The glandular epithelial cells of the Henle’s crypts (green arrows) showed some staining that increased within the extracellular, luminal part. Antibodies TMPRSS2-ab109131 and TMPRSS2-HPA035787 stained macrophages (purple arrows). The level of background staining may be estimated from the staining for ACTA2 and AIF1 that showed intense staining in the vascular media layer and macrophages, respectively, in addition to a weak background including Meibomian glands and epithelium.

**Figure 5:**
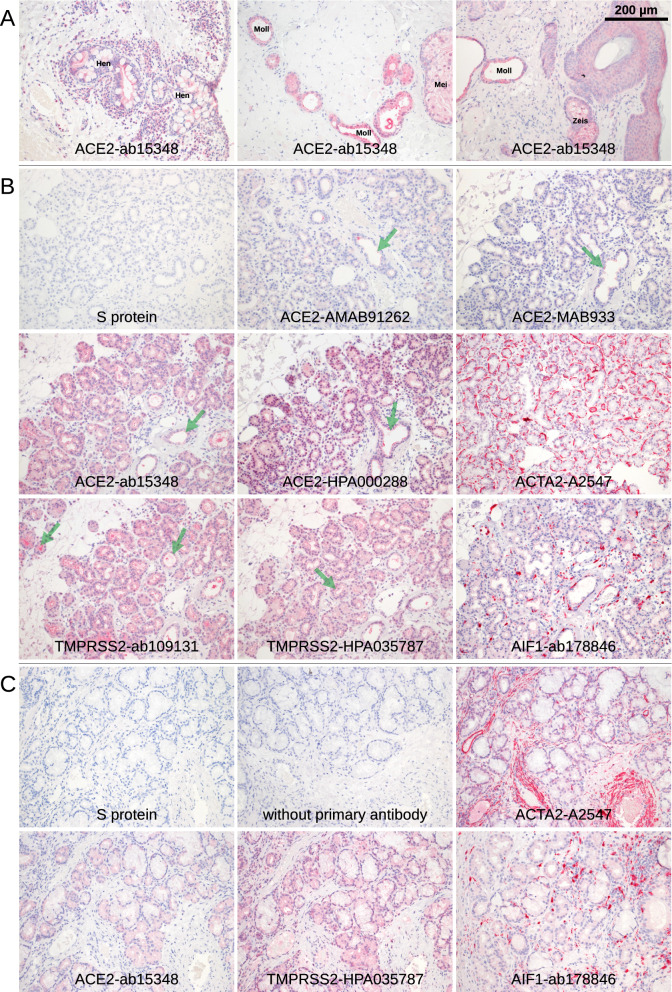
Eye lid, lacrimal gland, nose. Staining of serial paraffin sections (red). (**A**) The antibodies targeting ACE2 showed a week staining in the Meibomian glands (Mei), the Henle’s crypts (Hen) in the tarsal region, the Moll glands, and the Zeis sebaceous glands near the hair follicle. In all glands, there was a weak staining in the glandular epithelium that was more intense on the secretory side of the cells and thus adjacent to the glandular lumina. In addition, staining intensity increased in the distal parts of the tubes. (**B**) The lacrimal gland showed no staining for S protein or rarely some staining restricted to small areas. Week staining of the glandular tissue was found when stained with the antibodies, and it was also found with antibodies targeting ACTA2 and AIF1 indicating background. In contrast, a stronger, rather specific staining was at the luminal side of the glandular epithelium (green arrows). (**C**) Subepithelial glands within the skin of the nasal cavity showed staining for ACE2 and TMPRSS2 while S protein was negative. Staining for ACTA2 and AIF1 showed the same weak staining of the glands so that this staining is background rather than specific.

The lacrimal gland and several additional glandular structures in the lid and ocular surface tissue contribute to proper tear film formation. Similar to the ocular surface, no significant S protein staining was found in the lacrimal gland and eyelid glandular structures, including the Meibomian glands, Zeis' glands at the hair follicle, Moll's glands, Henle's crypts near the tarsal side of the lid and goblet cells at the tarsal side and nasal mucosa. Non-specific diffuse staining for ACE2 and TMPRSS2 was found in the lacrimal gland, as well as the tarsal and nasal mucosa (Figs. 4, 5, Table 2). Antibodies directed against ACE2 gave a positive signal in some tarsal epithelial regions, but no specific epithelial signal was detected for TMPRSS2 at these sites. Occasionally, the mucinous part of some glandular tubes or in rare cases individual goblet cells (1 sample out of 8) were intensely stained, indicating some expression of ACE2 in glandular structures.

At the RNA level, *ACE2* showed hardly any expression in all 6 lid specimens (median 0.8 TPM, IQR: 0.1–1.1, min 0.0 TPM, max 1.4 TPM). Similarly, lid samples showed hardly any expression of *TMPRSS2* (median 0.9 TPM, IQR 0.6–1.8 TPM, min 0.5 TPM, max 5.3 TPM) (Fig. 3). As a reference, the expression of the epithelial marker KRT10 (keratin 10) was 3385.0 TPM (IQR: 2159.0–6220.0), indicating significant expression of this marker in the samples studied (see Fig. 3A). In the lacrimal gland, no expression of *ACE2* was found in 8 specimens (all samples 0 TPM) and slightly higher expression levels for *TMPRSS2* (median 8.1 TPM, IQR 6.1–11.7 TPM) were detected (Fig. 3). As a reference, the median expression for *LTF* (lactotransferrin) was 10,509.0 TPM (IQR: 8703.0–12,321.0) indicating a significant expression of this marker in the samples analyzed (Suppl. Figure 3A). No expression of *ACE2* was found in three nasal mucosa specimens (all samples 0 TPM) which was associated with very low expression levels for *TMPRSS2* (median 12.9 TPM, IQR 8.3–14.3 TPM) (Fig. 3 and Suppl. Figure 3B). As a reference, the expression of *MUC5B* (mucin 5B) was 321.3 TPM (IQR: 255.0–649.1), indicating significant expression of this marker in the samples studied (see Fig. 3B).

Overall, the lacrimal gland and lid margins showed no or very weak S-protein and ACE2 staining, respectively, and barely detectable ACE2 mRNA expression, indicating a low probability of a relevant viral entry site for SARS-CoV-2.

### S protein and ACE2 immunoreactivity in intraocular tissue

**Figure 6:**
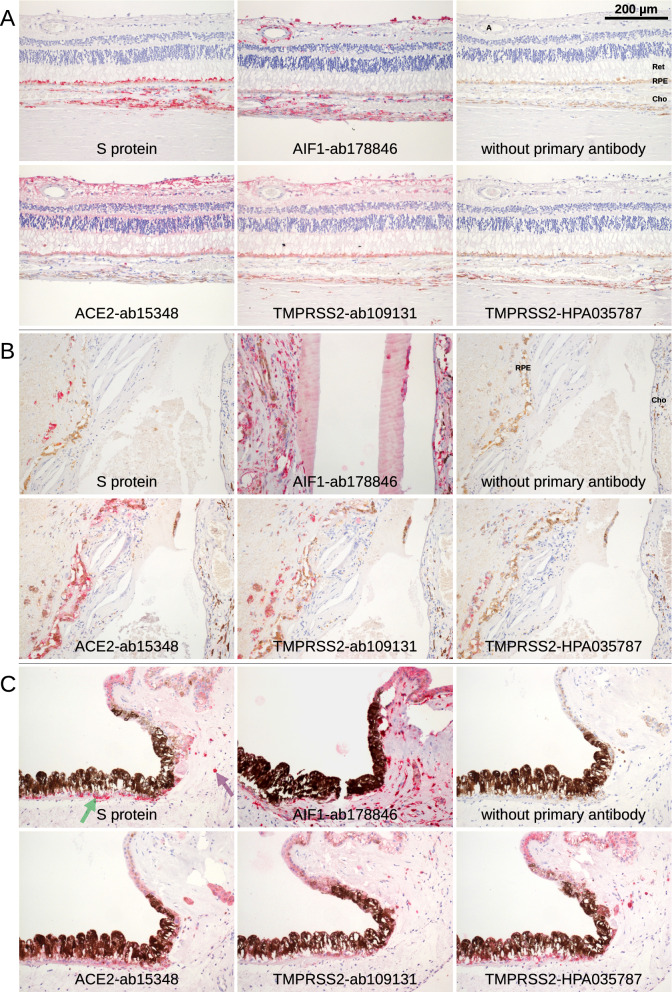
Retina, RPE, choroid, iris, ciliary body. Immunohistochemical staining of serial paraffin sections. (**A**) Retina (Ret), RPE, choroid (Cho), and sclera. Staining with S protein was restricted to RPE and choroid. Most cells in the choroid that were stained with S protein were pigmented. Staining for AIF1 (IBA1) identified them as macrophages. A similar pattern was found with the TMPRSS2 antibodies TMPRSS2-HPA035787 and TMPRSS2-ab109131. The latter showed an additional staining in the retina. The ACE2 antibodies showed weak staining or stained mainly the RPE and the retina. Sclera as well as the artery (**A**) in the retina were not stained. (**B**) Pigmented cells in subretinal fibrotic tissue (left) near the optic nerve head were stained with S protein. The line of brown cells is the remnant of the RPE, and the choroid at the right is separated by a large bleeding and fibrotic tissue. Antibodies for ACE2 and TMPRSS2 showed the same pattern. (**C**) Staining with S protein was found in the pigmented iris epithelium (green arrow) as well as in the epithelium of the ciliary body. In addition, some (pigmented) cells in the underlying tissue were stained (purple arrow). The cells were also positive for AIF1 indicating macrophages. The most similar pattern was observed with TMPRSS2-HPA035787, while TMPRSS2-ab109131 and ACE2-ab15348 also showed a comparable pattern. No or weak staining was found with the other antibodies.

In contrast to the ocular surface, lid, and lacrimal gland, iris and ciliary body specimens revealed some staining with S protein in 11 of 19 analyzed specimens (Fig. 6 and Suppl. Figure 4, Table 2). Staining was found in the pigmented iris epithelium, the epithelium of the ciliary body, or in pigmented cells of the stromal tissue of the iris. Staining for AIF1 indicated that most of the cells that were stained with S protein are macrophages.

Similarly, staining with S protein was found in the RPE and to a lesser extent in the choroid whereas no signal was detected in the retina and sclera (Fig. 6 and Suppl. Figure 5). Staining in the choroid was mostly limited to pigmented cells, which may include choroidal melanocytes or macrophages. The choroidal staining pattern correlated with that of AIF1 indicating that mainly macrophages are labelled with S protein. The stained tissue sections were also positive for ACE2 and TMPRSS2. Staining for TMPRSS2 was rather similar to staining by bound S protein while staining for ACE2 was weak, in particular in the choroid. The retina was immunostained with antibodies ACE2-ab15348 or TMPRSS2-ab109131 and to a lesser extent with TMPRSS2-HPA035787 mainly in the ganglion cell layer (Fig. 6A).

In the retina, no *ACE2* expression (all samples 0 TPM) and hardly any expression of *TMPRSS2* (1 sample with 0.5 TPM) were detected at the RNA level in 6 retinal samples (Fig. 3). In RPE and choroid specimens, a low *ACE2* expression of 3.9 TPM could be detected in one sample, although the remaining 3 samples showed no *ACE2* expression. In addition, a low *TMPRSS2* expression of 4.7 and 3.4 TPM was measured in 2 of the 4 RPE and choroid samples. Analyzing the expression of *NRP1* revealed slightly higher levels in retinal (median 3.7 TPM, IQR 2.5–10.8 TPM) and in particular in choroid/RPE specimens (median 28.9 TPM, IQR 24.0–38.4 TPM). As a reference, the expression of *RHO* (rhodopsin) and *BEST1* (bestrophin 1) were analyzed in retinal and RPE/choroid samples, respectively. In retinal samples, the median expression of *RHO* was 6052.0 TPM (IQR: 1193.0–8115.0) and in RPE/choroid samples, the median expression of *BEST1* was 35.8 TPM (IQR: 16.5–103.0), respectively, indicating significant expression of these marker in the samples analyzed (Suppl. Figure 3B). The expression levels of various discussed (co-) receptors for SARS-CoV-2 cell entry, including *NRP1*, *FURIN* (furin), *BSG*, *DPP4* (dipeptidyl peptidase 4), *CTSL* (cathepsin L), *HSPA5* (heat shock protein family A member 5), *ANPEP* (alanyl aminopeptidase), *CEACAM1* (CEA cell adhesion molecule 1), *CD209* and *CLEC4M* (C-type lectin domain family 4 member M) are visualized in Suppl. Figure 3.

The optic nerve samples showed hardly any expression of *TMPRSS2* (one sample with 0.3 TPM) (Fig. 3). As a reference, the expression of the optic nerve marker *AQP4* was analyzed. The median expression for *AQP4* (aquaporin 4) was 491.2 TPM (IQR: 365.3–652.7) indicating a significant expression of this marker in the samples analyzed (Suppl. Figure 3B). At the RNA level, *ACE2* was not detectable in all 4 optic nerve specimens (all samples 0 TPM). Similarly, the optic nerve was not stained with S protein.

## Discussion

It is generally accepted that COVID-19 patients rarely exhibit clinical signs of ocular involvement, a finding that may be explained by the reported low expression of SARS-CoV-2 entry sites including ACE2 and TMPRSS^16^. However, there is an ongoing discussion and some clinical evidence pointing towards a possible ocular involvement in some COVID-19 patients^4^. In the study at hand, we therefore investigated possible entry sites for SARS-CoV-2 by using biotin-labeled recombinant viral S protein for histochemistry in different ocular tissues and compared the staining pattern with RNA as well as protein expression of TMPRSS2 and ACE2. Our study demonstrates only little S protein binding and thus indicates scarcity of SARS-CoV-2 entry sites in various ocular surface tissues examined. Similarly, only weak concordant ACE2 or TMPRSS2 expression was found in extra- and intraocular tissues, which may explain the overall rare ocular involvement in COVID-19 patients.

SARS-CoV-2 uses ACE2 as its main receptor to enter cells. Accordingly, tissues expressing ACE2 are more susceptible to infection. ACE2 expression is usually detected by immunohistochemical approaches using antibodies directed against ACE2. However, the published results on ACE2 expression in the eye vary widely depending on antibodies used, their concentration, and different protocols for antigen retrieval in FFPE sections. Thus, some reports suggest a strong and ubiquitous presence of ACE2 in conjunctival epithelium, some propose an ACE2 signal confined to individual epithelial cells in distinct areas^12,14,15^, whereas others argue for a negligible presence of SARS-CoV-2 entry sites on the ocular surface^16^. As an alternative approach to detect possible binding sites of SARS-CoV-2, biotin-labelled recombinant viral S protein binding to ACE2 may be used for staining. The affinity of S protein binding to ACE2 (K_d_ = 1.2 nM)^8^ is in the same range as that of antibodies binding to their substrates^26,27^. Therefore, a histochemical staining within the same intensity range as is known from antibody staining can be expected. In the present study, we observed S protein staining signals comparable to staining signals obtained with anti-ACE2 and anti-TMPRSS2 antibodies. Though the crystal structure of TMPRSS2 has not yet been resolved, interaction of S protein with TMPRSS2 is predicted to occur at the cleavage sites of S protein at Arg685/Ser686 and Arg815/Ser816^28^ which is outside of the receptor binding domain for ACE2 that was used in our study. This makes it unlikely but does not exclude the possibility that staining with the receptor binding domain of S protein also detects TMPRSS2. Additional SARS-CoV-2 receptors may be stained, too.

Some staining with S protein was found in the RPE and in the choroid as well as in the ciliary body and the iris. Typically, the staining was restricted to pigmented cells found in the RPE, the choroid and iris which may include melanocytes and macrophages. These cells also showed staining for AIF1 indicating that they were macrophages. Of note, staining was restricted to some areas of the eye while others were not stained, indicating local expression of these markers and refuting global expression of SARS-CoV-2 entry sites in the posterior segment of the eye. Staining with antibodies to TMPRSS2 showed comparable patterns to ACE2 staining, while staining in the choroid with antibodies for ACE2 was less intense. The most prominent difference was in the retina that was not stained by S protein while antibodies ACE2-ab15348 and TMPRSS2-ab109131 resulted in a strong and diffuse retinal staining^29^. No or low expression of ACE2 in the retina was reported in rat^30,31^. Interestingly, expression of ACE2 in the RPE or the choroid was not reported in these studies. In lung tissue, we found ACE2 and TMPRSS2 mainly expressed in alveolar macrophages in accordance with the report of Song^32^ while Ortiz^33^ found it in a small fraction of type II alveolar cells but not in alveolar macrophages.

Binding of the S protein was barely detected in the cornea and conjunctiva and associated with a general low expression of ACE2 and TMPRSS2 in the ocular surface. This finding indicates a low likelihood of SARS-CoV-2 infection via the ocular surface and may explain the reported low incidence of conjunctivitis in COVID19 patients^34^. Furthermore, the low S protein binding in the lacrimal gland and glands of the lid suggests that these glands are probably not very susceptible to SARS-CoV-2 infection following direct or indirect infection of the glands in the course of possible viremia. This finding may explain the clinical observation that SARS-CoV-2 virus RNA is rarely found in the tear film^34,35^. Moreover, it was observed that SARS-CoV-2, in contrast to HSV1 and Zika virus, did not replicate in isolated human corneal tissue^36^. The very limited expression of S protein binding proteins in ocular tissues was supported by our RNA-Seq experiments which only showed an insignificant *ACE2* transcription in 3 out of a total of 63 analyzed tissue which is in line with some earlier reports by other groups^17,18^. It may be questioned if such a low ACE2 expression is relevant for SARS-CoV-2 infection. It has to be noted, that some occasional expression of ACE2 immunoreactivity was detected in the ocular surface and lacrimal system, including a small spot in the limbal epithelium of a single conjunctiva similar to an earlier report^37^ and some parts of glandular tissue of some lacrimal gland specimens that showed weak staining. Although there is few evidence so far, it remains to be clarified whether individual factors such as genetic predisposition, inflammation or hypoxia can trigger ACE2 expression in the ocular surface and may be responsible for the observed occasional and localised ACE2 expression^10,12,29,38^.

SARS-CoV-2 may also enter cells by other receptors such as BSG (CD147)^39^ which may explain differences in the staining pattern of S protein and antibodies against ACE2. However, the affinity of S protein to BSG (K_d_ = 185 nM) is about 100 fold lower than that to ACE2 (high K_d_ means low affinity). In addition, the ocular expression pattern of BSG is different from the S protein staining pattern. BSG was reported to be expressed in the cornea^40^ where S protein was never found. The same applies for the retina^41^. Nevertheless, it cannot be excluded that BSG is up-regulated in certain conditions such as corneal ulceration^42^ were it may function as a receptor for SARS-CoV-2 viral entry. SARS-CoV-2 was detected by PCR on the ocular surface^3,6,43^. This method does not clarify if the virus is still infectious or already non-functional. In addition, PCR is a very sensitive method that may detect amounts of SARS-CoV-2 that are not high enough to result in a successful infection.

In summary, this study by S-protein histochemistry showed hardly any SARS-CoV-2 entry sites in all ocular tissues examined. Similarly, only weak ACE2 or TMPRSS2 expression was found in extra- and intraocular tissues. While this study suggests a rather low risk of ocular infection with SARS-CoV-2, it should be noted that potential viral entry sites may increase in response to inflammation or in certain disease states.

## Supplementary Information

Supplementary Information.

## References

[1] Barnett BP (2020). Potential of ocular transmission of SARS-CoV-2: A review. Vis. Basel Switz..

[2] Inomata T (2020). Clinical and prodromal ocular symptoms in coronavirus disease: A systematic review and meta-analysis. Invest. Ophthalmol. Vis. Sci..

[3] La Distia Nora R (2020). Are eyes the windows to COVID-19? Systematic review and meta-analysis. BMJ Open Ophthalmol..

[4] Chen Z, Yuan G, Duan F, Wu K (2020). Ocular involvement in coronavirus disease 2019: Up-to-date information on its manifestation, testing, transmission, and prevention. Front. Med..

[5] Sopp NM, Sharda V (2021). An eye on COVID-19: A meta-analysis of positive conjunctival reverse transcriptase-polymerase chain reaction and SARS-CoV-2 conjunctivitis prevalence. Optom. Vis. Sci. Off. Publ. Am. Acad. Optom..

[6] Qu J-Y, Xie H-T, Zhang M-C (2021). Evidence of SARS-CoV-2 transmission through the ocular route. Clin. Ophthalmol. Auckl. NZ.

[7] Hoffmann M (2020). SARS-CoV-2 cell entry depends on ACE2 and TMPRSS2 and is blocked by a clinically proven protease inhibitor. Cell.

[8] Walls AC (2020). Structure, function, and antigenicity of the SARS-CoV-2 spike glycoprotein. Cell.

[9] Hartenian E (2020). The molecular virology of coronaviruses. J. Biol. Chem..

[10] Collin J (2021). Co-expression of SARS-CoV-2 entry genes in the superficial adult human conjunctival, limbal and corneal epithelium suggests an additional route of entry via the ocular surface. Ocul. Surf..

[11] Grajewski RS (2021). A missing link between SARS-CoV-2 and the eye?: ACE2 expression on the ocular surface. J. Med. Virol..

[12] Li S (2021). SARS-CoV-2 receptor ACE2 is expressed in human conjunctival tissue, especially in diseased conjunctival tissue. Ocul. Surf..

[13] Mencucci R (2021). Co-expression of the SARS-CoV-2 entry receptors ACE2 and TMPRSS2 in healthy human conjunctiva. Exp. Eye Res..

[14] Roehrich H, Yuan C, Hou JH (2020). Immunohistochemical study of SARS-CoV-2 viral entry factors in the cornea and ocular surface. Cornea.

[15] Zhou L (2020). ACE2 and TMPRSS2 are expressed on the human ocular surface, suggesting susceptibility to SARS-CoV-2 infection. Ocul. Surf..

[16] Lange C (2020). Expression of the COVID-19 receptor ACE2 in the human conjunctiva. J. Med. Virol..

[17] Leonardi A, Rosani U, Brun P (2020). Ocular surface expression of SARS-CoV-2 receptors. Ocul. Immunol. Inflamm..

[18] Xiang M (2019). Comparative transcriptome analysis of human conjunctiva between normal and conjunctivochalasis persons by RNA sequencing. Exp. Eye Res..

[19] Boneva S (2020). 3’ MACE RNA-sequencing allows for transcriptome profiling in human tissue samples after long-term storage. Lab. Investig. J. Tech. Methods Pathol..

[20] Jalili V (2020). The Galaxy platform for accessible, reproducible and collaborative biomedical analyses: 2020 update. Nucleic Acids Res..

[21] Dobin A (2013). STAR: Ultrafast universal RNA-seq aligner. Bioinforma. Oxf. Engl..

[22] Liao Y, Smyth GK, Shi W (2014). featureCounts: an efficient general purpose program for assigning sequence reads to genomic features. Bioinforma. Oxf. Engl..

[23] Wagner GP, Kin K, Lynch VJ (2012). Measurement of mRNA abundance using RNA-seq data: RPKM measure is inconsistent among samples. Theory Biosci. Theor. Den Biowissenschaften.

[24] Yates AD (2020). Ensembl 2020. Nucleic Acids Res..

[25] Wickham H (2016). ggplot2: Elegant Graphics for Data Analysis.

[26] Pan Y (2016). Determination of equilibrium dissociation constants for recombinant antibodies by high-throughput affinity electrophoresis. Sci. Rep..

[27] Zhang T, Nagel-Steger L, Willbold D (2019). Solution-based determination of dissociation constants for the binding of Aβ42 to antibodies. ChemistryOpen.

[28] Hussain M (2020). Molecular docking between human TMPRSS2 and SARS-CoV-2 spike protein: Conformation and intermolecular interactions. AIMS Microbiol..

[29] Zhou L (2021). Expression of the SARS-CoV-2 receptor ACE2 in human retina and diabetes—implications for retinopathy. Invest. Ophthalmol. Vis. Sci..

[30] Foureaux G (2015). Activation of endogenous angiotensin converting enzyme 2 prevents early injuries induced by hyperglycemia in rat retina. Braz. J. Med. Biol Res. Rev. Bras. Pesqui. Medicas E Biol..

[31] Tikellis C (2004). Identification of angiotensin converting enzyme 2 in the rodent retina. Curr. Eye Res..

[32] Song J (2021). Distinct effects of asthma and COPD comorbidity on disease expression and outcome in patients with COVID-19. Allergy.

[33] Ortiz ME (2020). Heterogeneous expression of the SARS-Coronavirus-2 receptor ACE2 in the human respiratory tract. EBioMedicine.

[34] Al-Sharif E (2020). Ocular tropism of coronavirus (CoVs): A comparison of the interaction between the animal-to-human transmitted coronaviruses (SARS-CoV-1, SARS-CoV-2, MERS-CoV, CoV-229E, NL63, OC43, HKU1) and the eye. Int. Ophthalmol..

[35] Xia J, Tong J, Liu M, Shen Y, Guo D (2020). Evaluation of coronavirus in tears and conjunctival secretions of patients with SARS-CoV-2 infection. J. Med. Virol..

[36] Miner JJ (2020). HSV-1 and zika virus but Not SARS-CoV-2 replicate in the human cornea and are restricted by corneal type III interferon. Cell Rep..

[37] Eriksen AZ (2021). SARS-CoV-2 infects human adult donor eyes and hESC-derived ocular epithelium. Cell Stem Cell.

[38] Ziegler CGK (2020). SARS-CoV-2 receptor ACE2 is an interferon-stimulated gene in human airway epithelial cells and is detected in specific cell subsets across tissues. Cell.

[39] Wang K (2020). CD147-spike protein is a novel route for SARS-CoV-2 infection to host cells. Signal Transduct. Target. Ther..

[40] Mauris J, Woodward AM, Cao Z, Panjwani N, Argüeso P (2014). Molecular basis for MMP9 induction and disruption of epithelial cell-cell contacts by galectin-3. J. Cell Sci..

[41] Ochrietor JD (2003). Retina-specific expression of 5A11/Basigin-2, a member of the immunoglobulin gene superfamily. Invest. Ophthalmol. Vis. Sci..

[42] Cruzat A (2018). Colocalization of galectin-3 with CD147 is associated with increased gelatinolytic activity in ulcerating human corneas. Invest. Ophthalmol. Vis. Sci..

[43] Azzolini C (2021). SARS-CoV-2 on ocular surfaces in a cohort of patients with COVID-19 from the lombardy region, Italy. JAMA Ophthalmol..

