# Supplementary material for: Viral S protein histochemistry reveals few potential SARS-CoV-2 entry sites in human ocular tissues
Source: Sci Rep. 2021 Sep 27;11:19140. doi: 10.1038/s41598-021-98709-y (PMC8476534; doi:10.1038/s41598-021-98709-y)

Viral S protein histochemistry reveals few potential SARS-CoV-2 entry sites in human ocular tissues

Gottfried Martin, Julian Wolf, Thabo Lapp, Hansjürgen T. Agostini, Günther Schlunck, Claudia Auw-Hädrich, Clemens A. K. Lange

Eye Center, Medical Center, Medical Faculty, University of Freiburg, Freiburg, Germany

Suppl. fig. 1. Serial dilution of antibodies.

Serial paraffin sections were stained red with different concentrations of antibodies raised against ACE2 or TMPRSS2 while ACTA2 and VWF were used as examples for specific staining.

A: Conjunctiva. VWF is expressed in the endothelium of blood vessels (green arrow). While staining of the vessels is rather constant at a large range of dilutions, background in the epithelium or the subepithelial tissue is not. The same is true for ACTA2 that is expressed in the tunica media of arteries. In contrast, there is no such constant pattern in the stainings with antibodies for ACE2 or TMPRSS2. Therefore, it is estimated to consist largely of background staining. ACE2-AMAB91262 shows little signal and little background. From this, it is concluded that this conjunctival specimen shows no specific expression of ACE2 or TMPRSS2.

B: Eye lid. VWF and ACTA2 show specific patterns over a large range of concentrations while background is changing. Similarly, the tubes of the Henle's crypts (green arrows) show a specific pattern in contrast to changing background. Meibomian glands (Mei), Henle's crypts (Hen), epithelium (Epi).

C: lacrimal gland. VWF and ACTA2 show specific patterns over a large range of concentrations while background is changing. No such specific staining was observed with antibodies targeting ACE2 or TMPRSS2.

Suppl. fig. 2. Optimization of S protein staining.

A: Paraffin sections of lung stained with S protein (red). The blocking step was with different concentrations of BSA or with Ultrablock. 2 % BSA was used in further experiments.

B: Staining with S protein but without addition of BSA to block unspecific binding. Paraffin sections from conjunctiva, eye lid, or lacrimal gland were used. While controls without S protein were negative, staining was found in muscles of the eye lid and some spots of undefined origin. In addition, there was some staining of the lacrimal gland and at the inner surface of glandular tubes. In all other stainings, 2 % BSA were added resulting in the disappearance of these stainings. It is generally accepted that some blocking is necessary to prevent unspecific staining but blocking is always associated with the risk of suppressing signal. This should be kept in mind when interpreting results.

Suppl. fig. 3. RNA-Seq.

Boxplots showing expression levels of all analyzed factors as well as tissue specific marker genes in all tissue types. A total of 63 ocular tissue specimens was analyzed. Each dot represents one sample. Number of specimens: healthy conjunctiva: 8, diseased conjunctiva: 34, lacrimal gland: 8, nasal mucosa: 3, retina: 6, RPE/choroid: 4. ACE2: Angiotensin I Converting Enzyme 2, TMPRSS2: Transmembrane Serine Protease 2, NRP1: Neuropilin 1, FURIN: Furin, Paired Basic Amino Acid Cleaving Enzyme, BSG: Basigin (Ok Blood Group), DPP4: Dipeptidyl Peptidase 4, CTSL: Cathepsin L, HSPA5: Heat Shock Protein Family A (Hsp70) Member 5, ANPEP: Alanyl Aminopeptidase, Membrane, CEACAM1: CEA Cell Adhesion Molecule 1, CD209: CD209 Molecule, CLEC4M: C-Type Lectin Domain Family 4 Member M, KRT19: Keratin 19, LTF: Lactotransferrin, RHO: Rhodopsin, BEST1: Bestrophin 1.

Suppl. fig. 4. Staining of iris and ciliary body with S protein or different antibodies.

Serial paraffin sections were stained red with S protein or different antibodies raised against ACE2 or TMPRSS2.

A: Connection between iris and ciliary body. Cells of the iris stroma, presumably macrophages, were stained with S protein. The antibodies showed similar pattern.

B: Staining with S protein or antibodies of iris tissue shows large pigmented macrophages.

C: Connection between iris and ciliary body. Pigmented cells of the iris stroma and the surrounding tissue were stained with S protein. The antibodies showed similar pattern.

Suppl. fig. 5. Staining of retina, RPE, and choroid with S protein or different antibodies.

Serial paraffin sections were stained red with S protein or different antibodies raised against ACE2 or TMPRSS2.

A: Staining with S protein in pigmented cells of the choroid. The same pattern was found with TMPRSS2-HPA035787 while TMPRSS2-ab109131 and ACE2-ab15348 additionally stained the retina (top) and the optic nerve (left bottom).

B: Staining of RPE and choroid with S protein or antibodies.

C: Staining of RPE and choroid with S protein or antibodies. Macrophages in the choroid.

Suppl. fig. 1A. Serial dilution of antibodies. Conjunctiva.

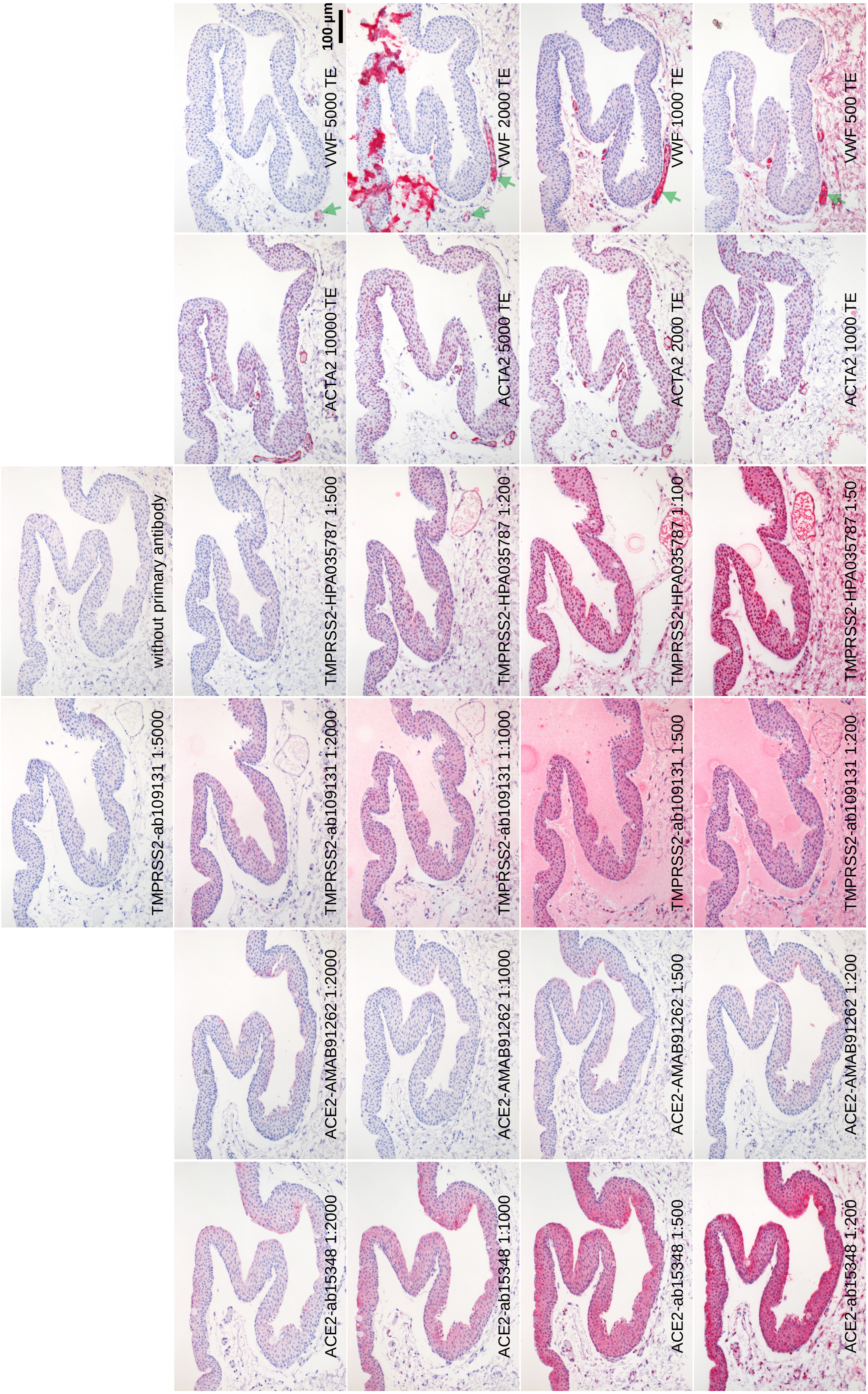

Suppl. fig. 1B. Serial dilution of antibodies. Eye lid.

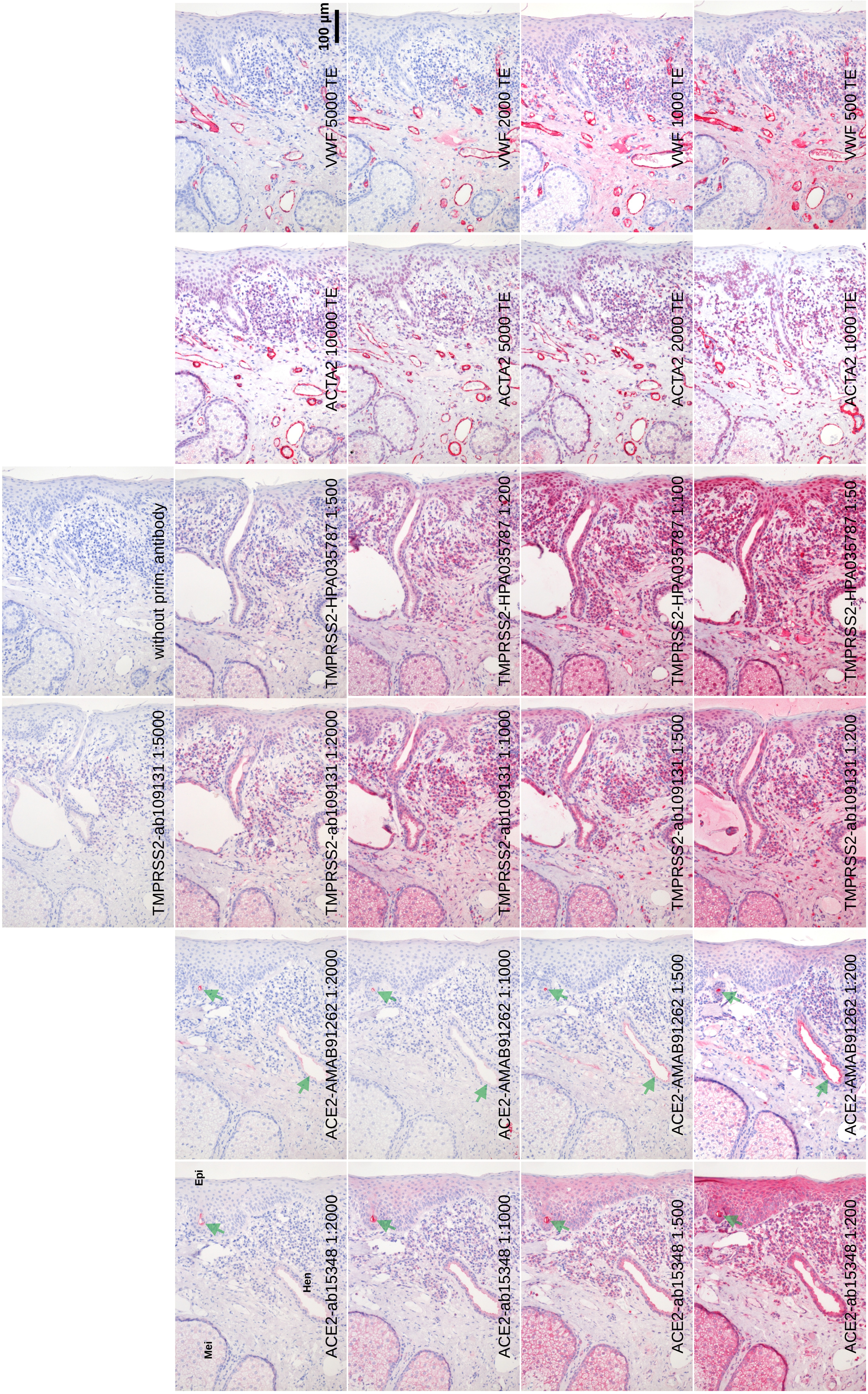

Suppl. fig. 1C. Serial dilution of antibodies. Lacrimal gland.

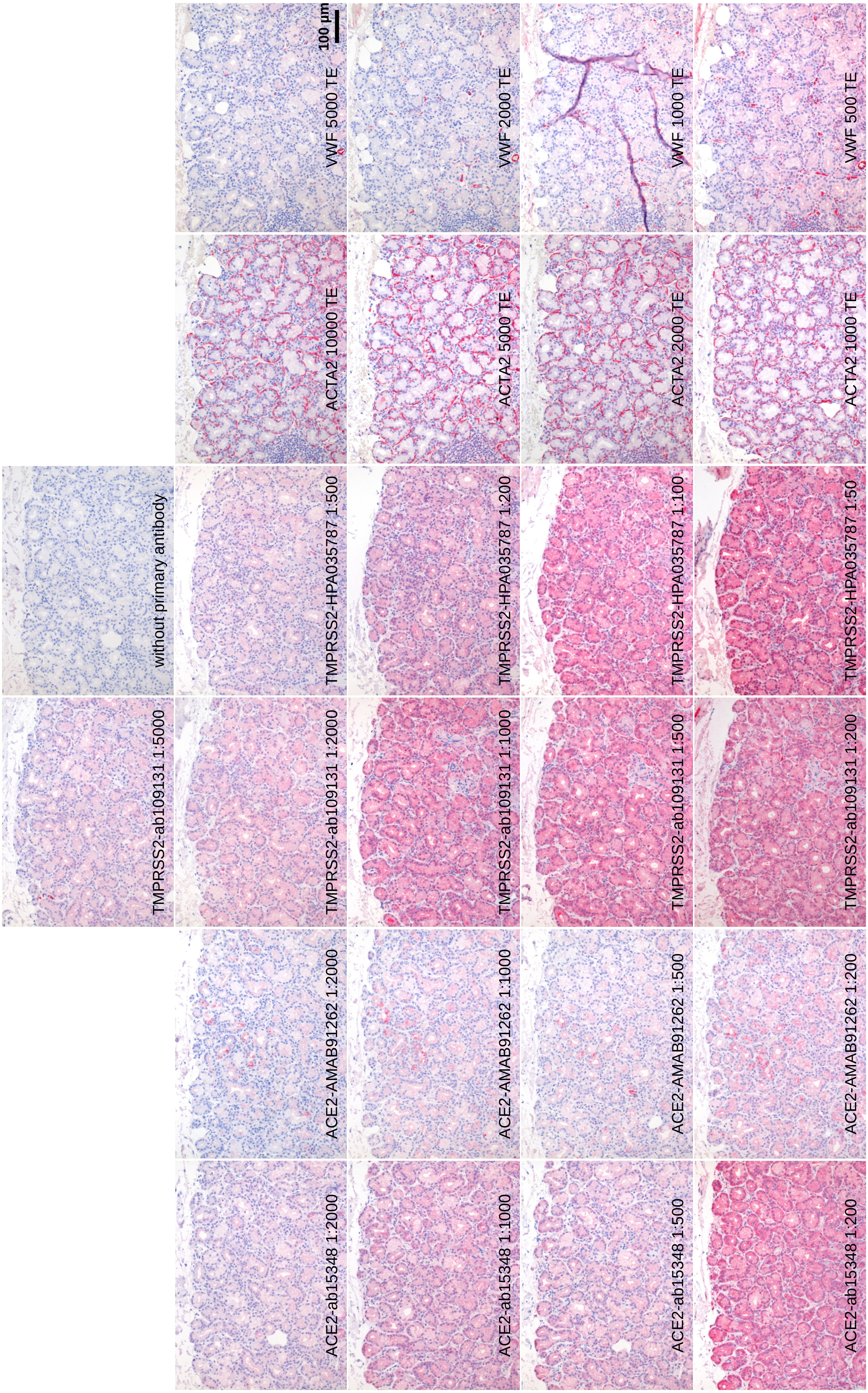

Suppl. fig. 2. Optimization of S protein staining.

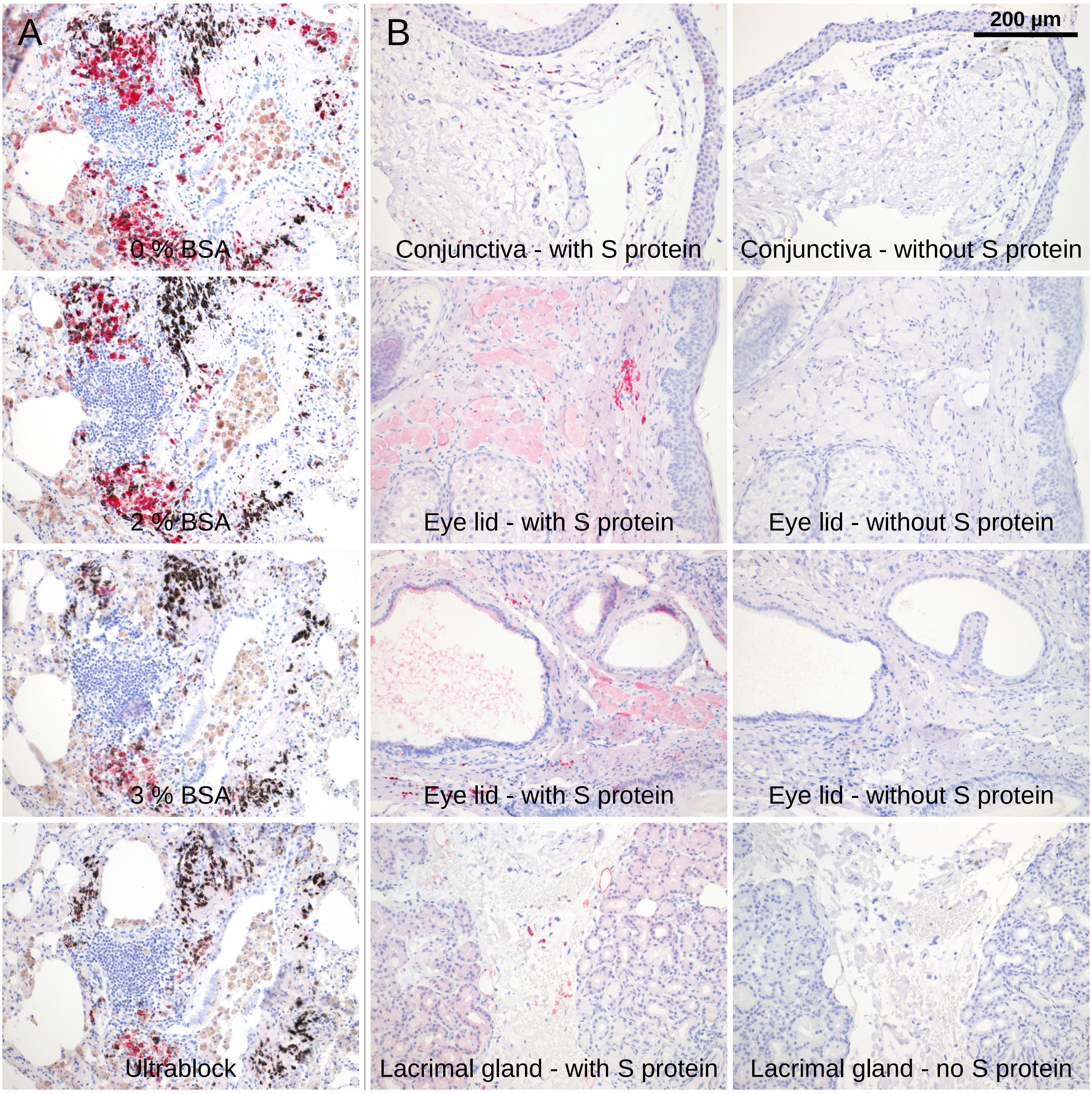

Suppl. fig. 3A. RNA-Seq.

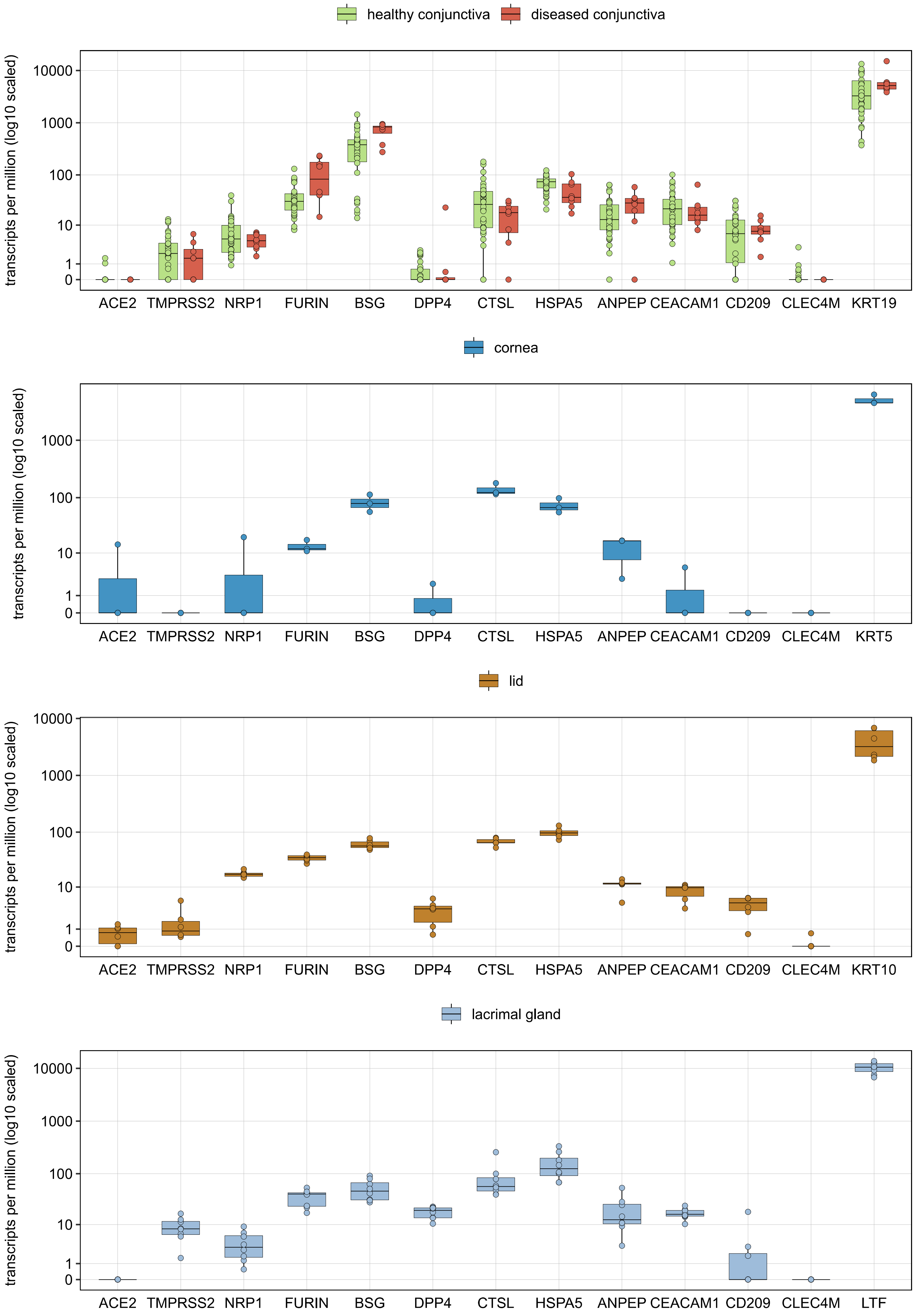

Suppl. fig. 3B. RNA-Seq.

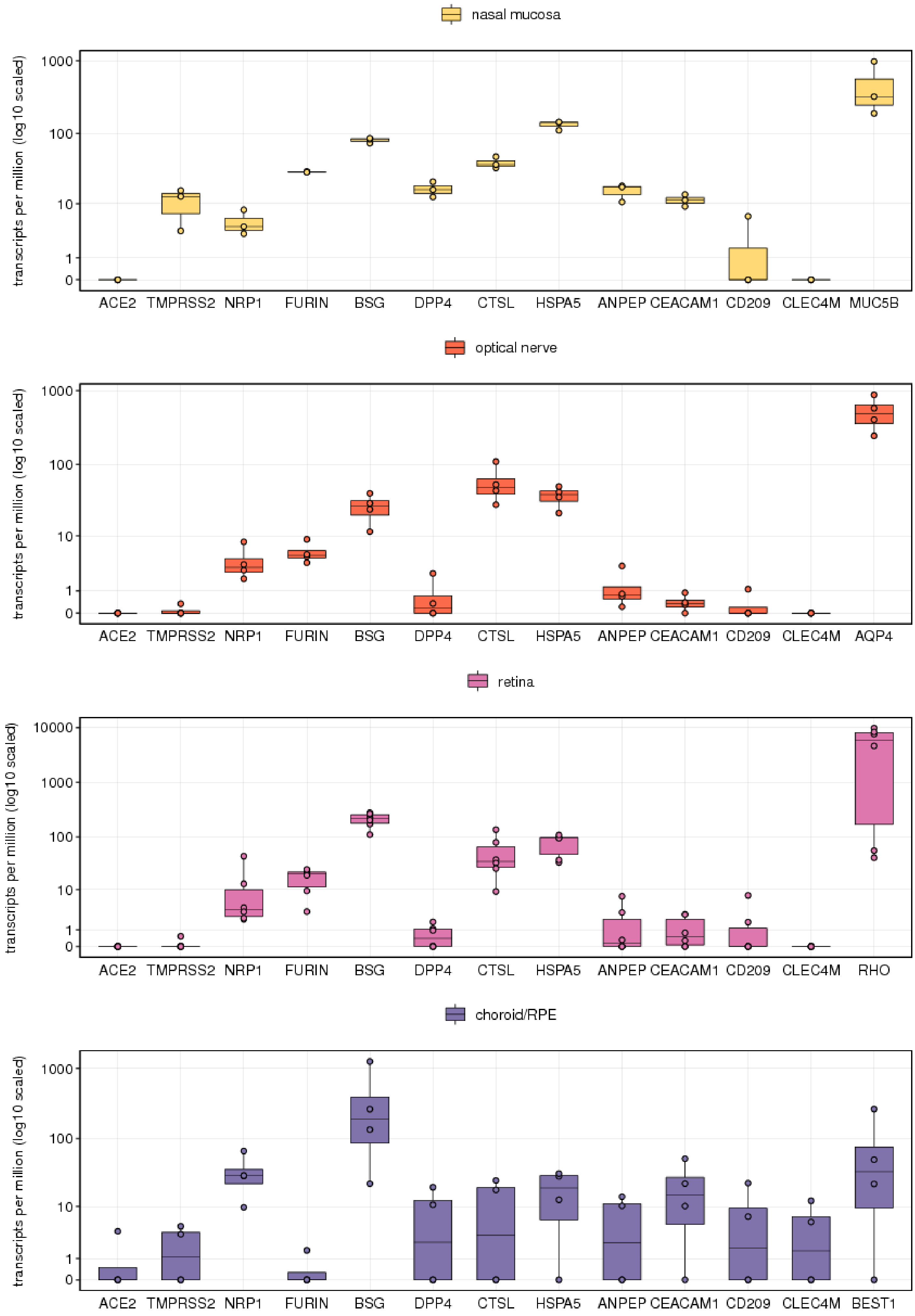

Suppl. fig. 4. Staining of iris and ciliary body with S protein or different antibodies.

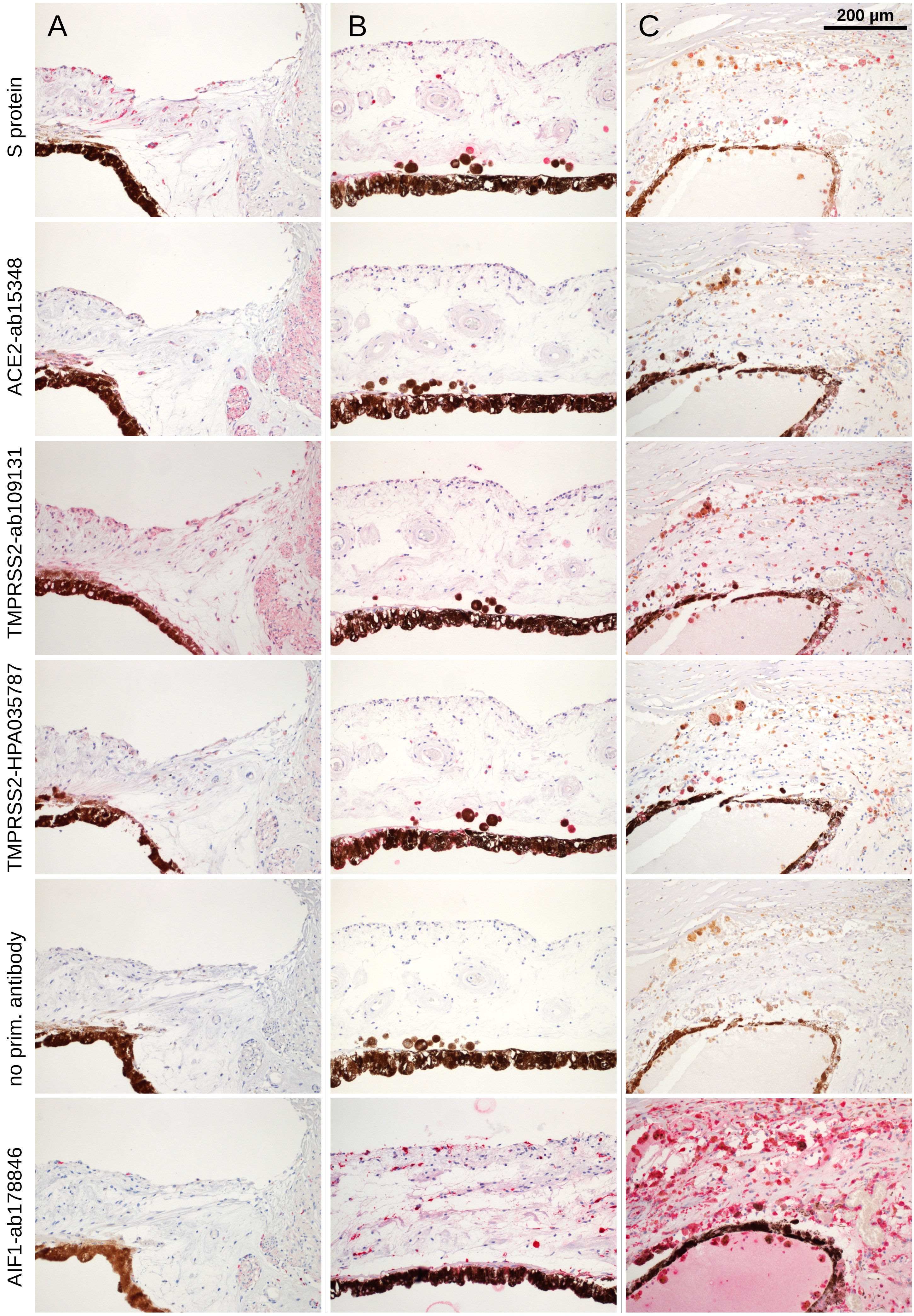

Suppl. fig. 5. Staining of retina, RPE, and choroid with S protein or different antibodies.

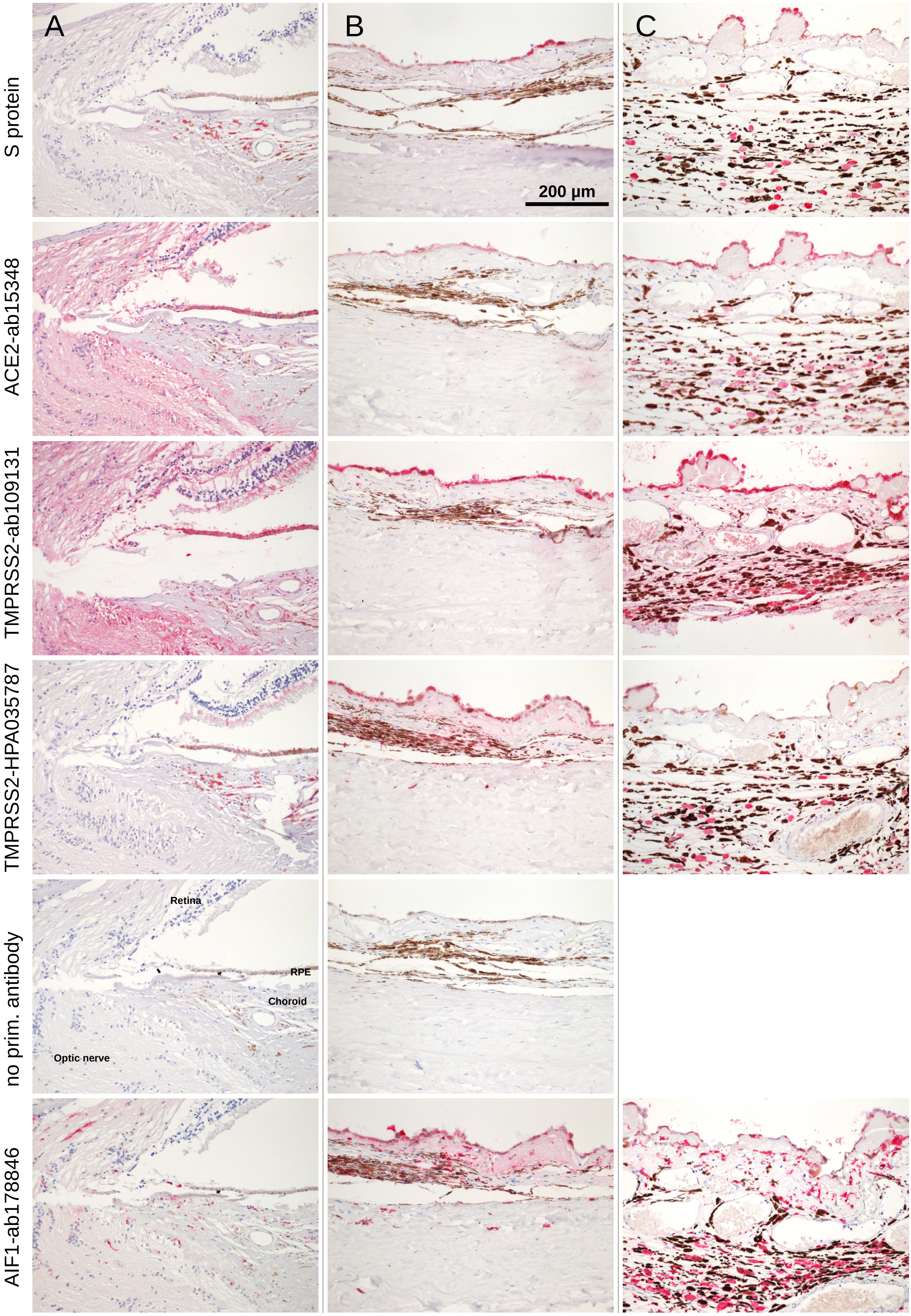

Supplement: Supplementary file 1 — Supplementary Information. [file 41598_2021_98709_MOESM1_ESM.pdf]
